# Comparison of virtual reality and physical simulation training in first‐year radiography students in South America

**DOI:** 10.1002/jmrs.639

**Published:** 2022-12-11

**Authors:** David Rowe, Alejandra Garcia, Benito Rossi

**Affiliations:** ^1^ University of Chile Clinical Hospital Independencia Región Metropolitana Chile; ^2^ Clinica Alemana Vitacura Región Metropolitana Chile

**Keywords:** Radiography, random allocation, simulation training, students, virtual reality, X‐rays

## Abstract

**Introduction:**

The aim of this study was to comparatively evaluate the learning outcomes achieved by first‐year radiography students educated with either virtual reality (VR) simulation training or physical simulation training. The implementation of VR has been proposed to enhance learning in radiography students and provide a more effective and efficient approach to simulation. However, the learning outcomes achieved with this approach have not been widely investigated.

**Methods:**

Through stratified randomisation, 188 radiography students were allocated to one of two matched groups: a VR group (using Virtual Medical Coaching's Radiography simulation) and a physical simulation group (using Philips' X‐ray equipment). Both groups were taught 31 radiography views over one 25‐week semester. Both groups were assessed in an Objective Structured Clinical Examination (OSCE), using actors as patients in a physical X‐ray environment. Assessment was conducted by assigning objective count scores for five assessment criteria.

**Results:**

The VR group achieved shorter OSCE duration and fewer errors in moving equipment and patient positioning: these results were statistically significant (*P* < 0.00). There was no significant difference in the frequency of errors in radiographic exposure setting between the VR and the physical simulation group. The current findings concur with the limited number of published studies concerning VR simulation in radiography.

**Conclusions:**

The results of this study demonstrated superior effectiveness and efficiency in the VR group. This provides preliminary evidence to introduce VR simulation in the host institution and provide evidence that it may be possible to replace the use of physical simulation across other years of the degree. Further research investigating these possibilities is warranted.

## Graphical abstract

OSCE assessment results from student radiographers show a statistically significant difference exists between students trained using virtual reality (VR) simulation compared to students trained with traditional simulation. This was seen in the time to complete the OSCE, the number of movements of the X‐ray tube, the number of incorrect movements of the X‐ray tube and the number of errors in patient positioning. These findings suggest that VR simulation is superior to traditional physical simulation for teaching radiographic positioning in first‐year student radiographers.

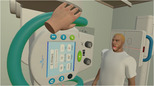

## Introduction

### Virtual reality simulation

Virtual reality (VR) is defined as the ‘use of computer technology to create the effect of an interactive three‐dimensional world, in which the objects have a sense of spatial presence’.^1^ Non‐immersive virtual experiences are created with a computer, monitor and input devices like keyboards, mice and controllers. Semi‐immersive virtual experiences offer a partial virtual environment, giving users the perception of being in a different environment when they focus on the display but also letting them remain connected to their surrounding environment.^2^ Fully immersive simulations offer the most realistic experience, complete with visuals, haptic feedback and audio sensations. The users need a head mount display (HMD) and hand controllers. HMDs provide high‐resolution content with a wide field of view and a stereoscopic 3D effect to establish an immersive, believable experience.^2^, ^3^ Haptic feedback allows the users to feel their environment.^4^ Audio is added to VR to further increase immersion. Two papers support the positive effects of a combined audio‐visual versus an exclusively visual VR environment due to audio enhancing the immersive nature of VR.^5^, ^6^ An example of this is an x‐ray tube can be heard running along the ceiling rails from one side of the room to the other.

### VR in healthcare education

Current literature discusses VR simulation as a cost‐effective and viable approach in healthcare education,^7^ with many papers emphasising the benefits of VR training in various surgical specialties. A systematic review investigating the effectiveness of VR in orthopaedic surgery reported an enhancement of surgical skills through training with VR.^8^ Similar findings were observed in a randomised control trial which evaluated the impact of VR surgery on the self‐perceived confidence and overall knowledge of surgical residents.^9^ Results demonstrated that the self‐perceived confidence of the VR cohort greatly improved when compared to the comparative group, and a vast improvement was seen in the overall knowledge of novice first‐year residents.^9^ VR for laparoscopic training has seen wide adoption with a due to its ability to lower error rates and operating times for novice surgeons.^10^, ^11^, ^12^

### VR in radiography education

In a recent study by Rainford and O'Connor VR learning was shown to improve the performance of first‐year students in their clinical assessments, particularly in the areas of patient positioning, exposure parameter selection, and image appraisal.^13^ Another study concluded that clinical placements are a key component of radiography education, supported by experienced clinical practice supervisors.^14^ Clinical placement in radiography is widely augmented by physical simulation‐based education, with a recent systematic review concluding that simulation is a valuable pedagogical approach for diagnostic radiography education.^15^ However, physical simulation requires access to a functioning or dummy X‐ray environment along with suitable physical phantoms for positioning and actual exposures, or actors as patients for positioning and non‐exposure simulation. While immersive VR simulation also demands a suitably equipped environment, the physical space required is less, and when the computers are turned off the virtual radiology room can be used for other purposes.^16^, ^17^, ^18^, ^19^ VR solutions allow users to conduct simulation outside of the traditional constraints of time and place, therefore removing the limitation of access to a simulation laboratory.^16^, ^20^ Clearly, all simulation‐based learning can carry a high cost,^21^ but the remote learning possibility with VR simulation does seem to offer advantages to learners. This is important in the light of an Irish study following the introduction of an immersive VR simulation tool in the curriculum: these authors reported over half the undergraduate radiography cohort requested more VR access in their learning.^16^

The adoption of VR technology in radiography education seems to have been accelerated in some centres during the COVID‐19 pandemic.^16^, ^22^, ^23^ One paper emphasises how home‐based learning negatively impacted on‐campus activities and how this created an opportunity for the expansion of VR in radiography education.^22^ Another suggests that VR simulation‐based education has specifically assisted students who were affected by lockdowns.^23^

### The current study

Despite the apparent adoption of VR simulation in health care, literature concerning its effectiveness in radiography education is limited. Studies of VR in radiographic education have primarily focused on student perceptions and willingness to incorporate the technology into their learning.^13^, ^16^, ^24^ For VR simulation to gain wide‐spread acceptance as a reliable pedagogical method in radiography, it must be shown to offer tangible benefits, in terms of learning outcomes, over less immersive or traditional teaching methods.^18^, ^25^ Investigations which focus on the achievement of competence in students trained with VR simulation are required. The current paper presents findings of a study which compared OSCE assessment outcomes between two matched cohorts of first‐year radiography students: one cohort trained with VR simulation and the other with physical simulation.

## Methods

### Ethical considerations

Institutional Ethics approval was granted to conduct this study (reference Acuerdo del comité de ética 2020/3487). The study was explained to first‐year radiography students in our university before they were invited to participate on a voluntary basis. Written consent was recorded for all student participants.

### Participant sample

A stratified random sampling approach was applied. The 188 volunteers from the first‐year student cohort were stratified by their prevailing grade average (above or below 70%) and by age (above or below 25 years), resulting in four strata. Students from each of the four strata were randomly allocated to two groups: 94 to the cohort who would learn with VR simulation and 94 to the cohort who would learn with physical simulation. The initial stratification allowed equivalence to be assumed between the two groups.

Both groups had the same previous experience in the degree programme. All the students had completed three two‐day clinical department visits as part of their degree orientation. They had observer time in general radiography, operating theatre, computed tomography, magnetic resonance imaging and nuclear medicine, with radiographic nurses in angiography, and accompanied porters. No student had operated any radiographic equipment.

### Study design

During the study, both groups were taught the same 31 radiographic views (see Table 1) and followed the anatomy, positioning and pathology semester timetable in the same order. The amount of simulation laboratory tuition time and tutorial time on campus was identical for both groups: 6 h per week for 25 weeks.

**Table 1:** Radiography views.

1	Cervical spine – odontoid peg
2	Cervical spine flexion and extension
3	Cervical spine anterior posterior
4	Cervical spine oblique – erect
5	Cervical spine – lateral
6	Chest Lateral
7	Chest Posteroanterior
8	Elbow Radial Head
9	Femur Anterior Posterior lower – supine
10	Femur Lateral ‐ lower (rolled)
11	Femur Lateral ‐ upper (horizontal ray)
12	Femur Lateral lower (horizontal ray)
13	Foot Weight‐bearing Dorsoplantar
14	Foot Weight‐bearing Lateral
15	Hip/Femur Anterior Posterior upper – supine
16	Knee Intercondylar notch
17	Knee anterior posterior
18	Knee lateral (rolled)
19	Knee Skyline (Laurin) knee
20	Pelvis Anteroposterior – Erect
21	Pelvis Judet – Iliac Oblique and Obturator Oblique
22	Lumbar spine Posteroanterior – Erect
23	Lumbar spine lateral – erect
24	Shoulder ACJ Anteroposterior – Erect
25	Shoulder ‐ Anterior Posterior – Erect
26	Shoulder ‐ lateral scapula ‐ Erect
27	Shoulder Axial ‐ seated
28	Shoulder Infero‐superior Axial – Supine
29	Facial bones Occipitomental
30	Facial bones Occipitomental 30°
31	Facial bones lateral

**Figure 1:**
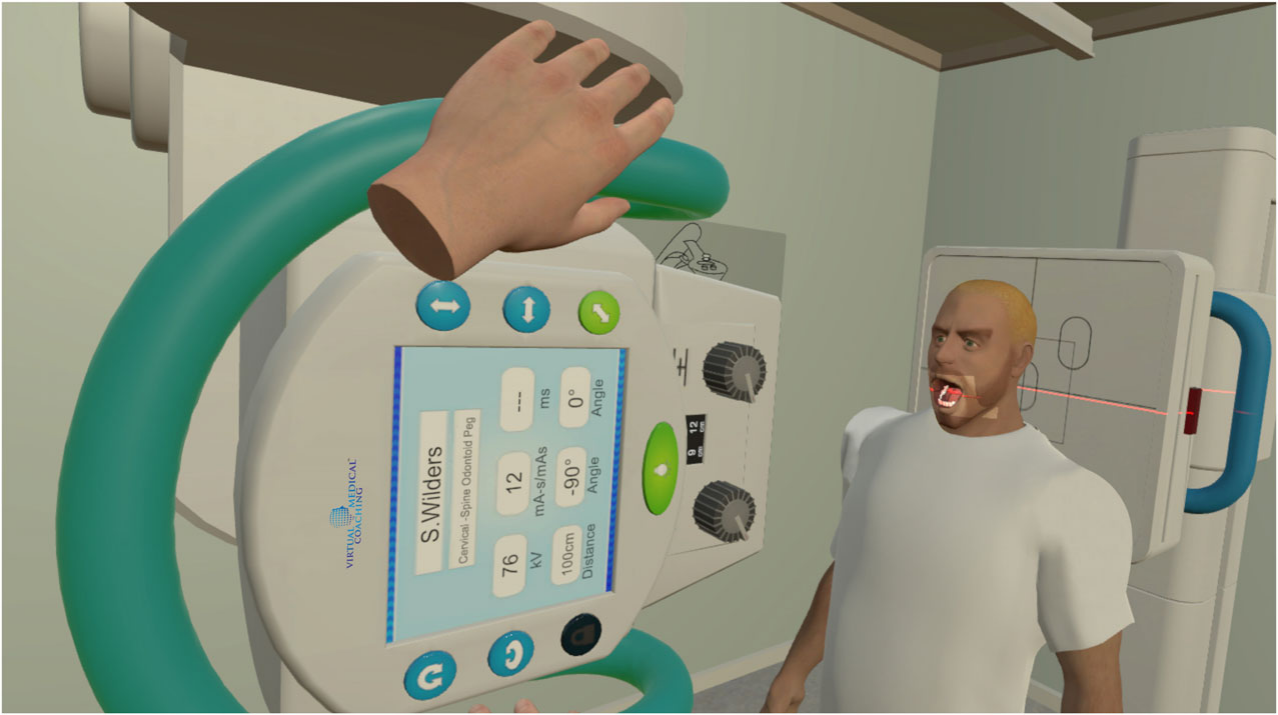
Student performing VR simulation.

Participants in the group assigned to VR simulation used Virtual Medical Coaching's radiography software for their simulation tutorial. See Figure 1 for an example of this. The learner wears a head set and holds hand controls to enter an immersive learning environment with audio, haptic and visual stimuli. The learner engages with a realistic radiography room and equipment and a selection of patients. The radiographic images produced by each learner reflect the patient and tube position and the selected exposure factors. The group were given training and time to familiarise themselves with the operation of the software.

Participants in the group assigned to physical simulation used physical equipment for their simulation tutorials. The equipment comprised a Phillips digital X‐ray room with a ceiling‐mounted X‐ray tube, erect detector and table, with a manikin for positioning. The group were given training and time to familiarise themselves with the operation of the equipment.

Following the 25‐week teaching and learning programme, the learning of both groups was examined in end of semester OSCE examinations.

The OSCE examinations involved physical X‐ray equipment and actors for patients. 39 student volunteers from a non‐healthcare degree with no ability to ‘help’ the learner under evaluation were employed to act as the patients. Each actor was given the same instructions to facilitate consistency. In the OSCE, each student was required to perform the same radiographic projections starting from the same initial environment in terms of X‐ray tube position, bed height, control panel settings and detector positions. Sixteen clinical radiographers with previous experience of assessing students in clinical departments evaluated the students during the OSCE. Neither the assessing radiographers nor the patient actors had any knowledge of whether a student was from the VR cohort or the physical simulation cohort, and the students themselves were instructed not to disclose this.

The OSCE assessment criteria were objectively specified to facilitate consistency across different assessors. The criteria groups were:

1. The duration of the OSCE
  The length of time each student took to perform the required projections was timed with a stopwatch.
2. Frequency of machinery movement
  This criterion evaluated movement of the X‐ray tube, table or detector in the x, y or z plane; rotation or angulation of the X‐ray tube or detector; and collimation opening, closing and field rotation. A movement was defined as any point where the student made contact with and moved the machinery until they had finished that specific movement.
3. Frequency of incorrect machinery movement
  This criterion also evaluated movement of the X‐ray tube, table or detector in the x, y or z plane; rotation or angulation of the X‐ray tube or detector; and collimation opening, closing and field rotation. Incorrect movement was defined as any movement of the machinery that the student reversed.
4. Frequency of radiographic exposure errors
  This criterion evaluated selection of Source to Image Distance (SID), kVp and mAs. Errors in radiographic exposure were defined as a selection that the assessor considered to be exposure settings that would create a diagnostic exposure. This was a difficult criterium to measure as with a digital system many errors in exposure will produce a diagnostic image, as will an incorrect SID.
5. Frequency of patient positioning errors
  This criterion evaluated the position of the body part under examination relative to the detector. Errors in patient positioning were defined as any position which the assessor would alter prior to exposure in a clinical setting.

The assessment criteria were measured and recorded in real time by the assessors using a tick box format form and an area for additional observations.

### Statistical analyses

**Figure 2:**
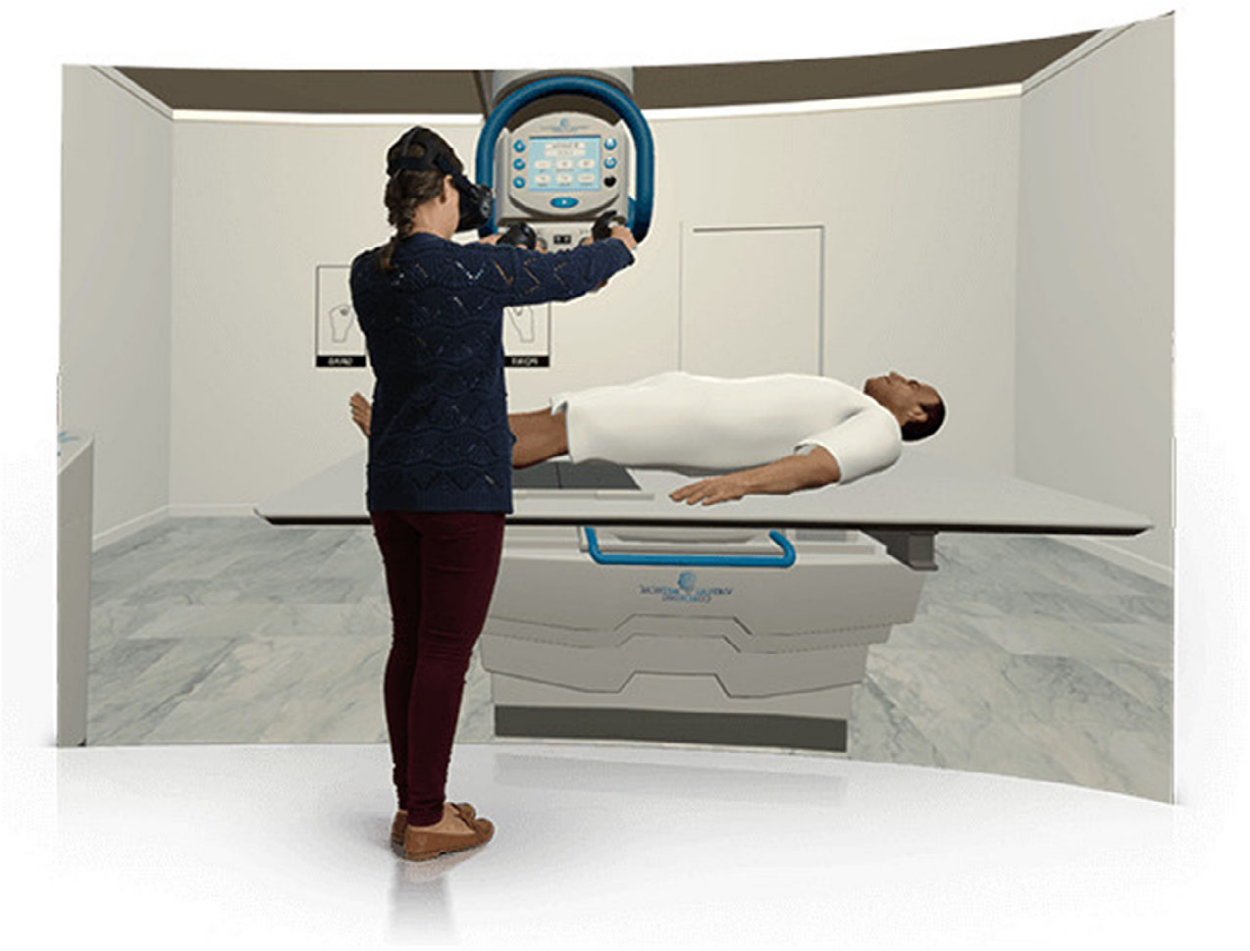
VR simulation in action.

The five assessment criteria were scored for each of the 31 projections each learner performed. The mean score for each assessment criterion was calculated for each learner and for each cohort: the VR simulation cohort and the physical simulation cohort. SPSS 26 (Statistical Package for Social Sciences) was used to run a Mann–Whitney U‐test to compare scores between the groups for each assessment criterion (Figure 2).

## Results

Descriptive statistics for each of the criterion scores are given in Table 2.

**Table 2:** Descriptive statistics.

Criterion	VR simulation cohort					Traditional physical simulation cohort				
	Mean	Min	Max	Range	Std. Deviation	Mean	Min	Max	Range	Std. Deviation
OSCE Time (Seconds)	912.245	688	1220	532.0	117.1908	961.47	711	1294	583.0	129.2289
Tube Movements (count)	29.837	16.0	47.0	31.0	6.3645	32.528	16.0	50.0	34.0	6.2092
Incorrect Movements (count)	3.606	0	8.0	8.0	2.1151	4.602	0	9.0	9.0	2.3664
Exposure Error (count)	1.006	0	2.0	2.0	0.8135	1.012	0	3.0	3.0	0.8204
Positioning Error (count)	0.917	0	4.0	4.0	1.0448	1.279	0	6.0	6.0	1.3786

Results of statistical comparison of the criterion scores of the VR simulation cohort and the physical simulation cohort are given in Table 3.

**Table 3:** Results of statistical comparison of the criterion scores of the VR simulation cohort and the physical simulation cohort.

Criterion	F Ratio	*P* Value	Interpretation
OSCE time	232.075	<0.000	OSCE time is significantly higher in the physical simulation cohort
Frequency tube movement	266.931	<0.000	Frequency of tube movement is significantly higher in the physical simulation cohort
Frequency incorrect movement	286.698	<0.000	Frequency of incorrect tube movement is significantly higher in the physical simulation cohort
Frequency exposure error	0.074	*P* = 0.785	No significant difference in frequency of exposure errors between cohorts
Frequency positioning error	127.654	<0.000	Frequency of positioning error is significantly higher in the physical simulation cohort

## Discussion

In line with published literature, the current results are positive for the use of VR simulation in radiography education. Students in the VR simulation cohort performed better than students in the physical simulation cohort on four out of five OSCE assessment criteria and at the same level on the fifth criterion (Table 2). An Australian study also focussing on first‐year student radiographers reported that after 3 weeks of training addressing posterior–anterior and oblique hand X‐ray positioning, learners who received VR education performed 36% better than those in the conventional simulation group in an examination on a real patient model.^26^ Another Australian study also compared outcomes from VR simulation and traditional laboratory‐based simulation, observing enhanced but not statistically significant technical skill acquisition in the VR simulation group compared with the traditional laboratory training group of medical imaging students.^27^ VR training seems to be as or more effective than more traditional training methods.

Until recently, physical simulation had been used in our programme to prepare students for clinical placement. In conjunction with literature reports, the current results provide reinforcement of the decision to introduce VR simulation in the host institution and provide evidence that it may be advantageous to replace the use of physical simulation. Such a decision could not be taken with certainty without extending the current study to investigate the use of VR simulation over physical simulation for students in later years of the degree programme, who would be learning more complex procedures. However, further adoption of VR simulation would convey multiple advantages. Training to safely use ionising radiation could be completed in a risk‐free environment. The practicality of being able to deploy VR software almost anywhere would offer students opportunity for repeated practice: this opportunity along with our results suggests that students could potentially be better prepared for clinical placements through VR simulation.

Virtual reality simulation also overcomes other limitations of traditional radiography simulation, such as limited availability of standardised patients and should VR simulation be extended to include assessment it can allay assessor fatigue throughout long assessment days.^28^ With VR simulation for assessment, assessment data are available in metric form, to be graded after the simulation or reviewed by assessors for grading at their own pace.

It is important to consider why students in the VR cohort were able to effectively transfer knowledge and skills from simulation to the real world. Clearly this arises from more than the ‘coolness’ factor of VR. The learning theory of Connectivism has been offered as a useful framework for understanding learning in the digital age.^20^, ^29^, ^30^, ^31^ Simply considered, Connectivism proposes that people process information by forming connections and that people do not stop learning after completing their formal lessons.^20^, ^32^ In VR simulation, immersion, presence and interactivity combine to offer users an engaging learning environment free from distractions that can be present in traditional simulation.^20^ During the immersive VR simulation, we used in this study, the X‐ray equipment and patients move and feel like those in a real environment. The realistic surroundings locate the student into a hospital‐like setting rather than a simulation laboratory environment where they have other potentially distracting stimuli such as their peers watching them. The HMD essentially closes the user's senses from the real world, so that the brain believes that the virtual world is real. The brain is engaged with the task and the neural connections needed for learning and memory are strengthened: that is, the conditions are ideal for learning and learning transfer as proposed under Connectivism.

### Strengths and limitations

The current study investigated difference in learning outcomes between radiography student cohorts learning with VR simulation and physical simulation. The two cohorts were well matched for previous learning experience and age and grade profile. The assessment criteria were objective and recorded in real time by experienced assessors, blind of the study cohort each student belonged to. These factors strengthen the findings of the comparison.

Multiple assessors scored the students in the OSCE, and no inter‐rater reliability evaluation was conducted. However, all assessors were qualified and experienced in grading students for their formal qualifications. While students were instructed not to cross over to another simulation style, there was no control over what they did outside of the dedicated learning environment. Some students may have accessed extra VR or physical simulation at home or on campus. These factors moderate the findings of the comparison.

The study was focussed on 1‐year group of students in one university, and while the findings are conclusively in favour of VR simulation, similar findings may not arise in later year groups in the same university or in student groups in other universities with different previous experience.

## Conclusion

The results suggest that widespread adoption of VR simulation in first‐year radiography education is timely and desirable. Our study adds to the evidence supporting the transition of simulation education in radiography from traditional to VR.

## Conflict of Interest

The authors declare no conflict of interest.
